# Centrifugal Inputs to the Main Olfactory Bulb Revealed Through Whole Brain Circuit-Mapping

**DOI:** 10.3389/fnana.2018.00115

**Published:** 2019-01-07

**Authors:** Krishnan Padmanabhan, Fumitaka Osakada, Anna Tarabrina, Erin Kizer, Edward M. Callaway, Fred H. Gage, Terrence J. Sejnowski

**Affiliations:** ^1^Crick-Jacobs Center for Theoretical and Computational Biology, Salk Institute for Biological Studies, La Jolla, CA, United States; ^2^Computational Neurobiology Laboratory, Salk Institute for Biological Studies, La Jolla, CA, United States; ^3^Department of Neuroscience, University of Rochester School of Medicine and Dentistry, Rochester, NY, United States; ^4^Systems Neurobiology Laboratories, Salk Institute for Biological Studies, La Jolla, CA, United States; ^5^Laboratory of Cellular Pharmacology, Graduate School of Pharmaceutical Sciences, Nagoya University, Nagoya, Japan; ^6^Laboratory of Genetics, Salk Institute for Biological Studies, La Jolla, CA, United States; ^7^Howard Hughes Medical Institute, Salk Institute for Biological Studies, La Jolla, CA, United States

**Keywords:** olfactory bulb, feedback, olfaction, retrograde tracer, circuits

## Abstract

Neuronal activity in sensory regions can be modulated by attention, behavioral state, motor output, learning, and memory. This is often done through direct feedback or centrifugal projections originating from higher processing areas. Though, functionally important, the identity and organization of these feedback connections remain poorly characterized. Using a retrograde monosynaptic g-deleted rabies virus and whole-brain reconstructions, we identified the organization of feedback projecting neurons to the main olfactory bulb of the mouse. In addition to previously described projections from regions such as the Anterior Olfactory Nucleus (AON) and the piriform cortex, we characterized direct projections from pyramidal cells in the ventral CA1 region of hippocampus and the entorhinal cortex to the granule cell layer (GCL) of the main olfactory bulb (MOB). These data suggest that areas involved in stress, anxiety, learning and memory are all tethered to olfactory coding, two synapses away from where chemical compounds are first detected. Consequently, we hypothesize that understanding olfactory perception, even at the earliest stages, may require studying memory and behavior in addition to studying the physiochemical features of odors.

## Introduction

Understanding the neural basis of olfactory perception remains one of the key challenges of sensory neuroscience. Part of the challenge arises from the inherently high dimensional nature of chemical structure (Secundo et al., 2014). For instance, mapping olfactory percepts onto any single feature of chemical structure such as carbon chain length, remains difficult. A parallel approach has focused on anatomy and physiology. In rodents, the neuronal representations of odors have been investigated by tracking neural activity from the olfactory receptor neurons (ORNs), where components of smell are first detected, to the piriform cortex (Stettler and Axel, 2009; Davison and Ehlers, 2011), where they are assembled combinatorially (Babadi and Sompolinsky, 2014) into odor percepts. However, such a feedforward model of olfactory coding, wherein neuronal responses are sequentially marshaled into more complex representations by progressively higher cortical areas glosses over the major feedback or centrifugal projections present in olfaction. As a result, comparatively less is known about the origin, identity, and organization of feedback projecting cells (Price and Powell, 1970; Shipley and Adamek, 1984).

To address this, we used a modified g-deleted rabies virus (Wickersham et al., 2007; Osakada and Callaway, 2013) and whole-brain imaging methods (Padmanabhan et al., 2010), to describe the organization and identity of centrifugal projections to the main olfactory bulb (MOB). In addition to confirming centrifugal inputs in the mouse that have been previously identified in the rat (Price and Powell, 1970), we also identified monosynaptic feedback from CA1 pyramidal cells in the hippocampus and from cells in the entorhinal cortex to the bulb. Our results provide a feedback-wiring diagram for the olfactory system of the mouse.

## Methods

Some of the data used in this manuscript has been previously published (Padmanabhan et al., 2016). In this pervious work, the focus was exclusively on projections from the AON and the piriform cortex, with the goal being to understand the organization of feedback circuits from these two olfactory regions. Here we provide a quantitative assessment of the organization of the feedback projections from the entire mouse brain. Additionally, this work uses analysis and provides information about the relative distribution of feedback projections that were not previously reported or quantified.

### Rabies Virus

GFP, mCherry, or BFP was cloned in pSADΔG-F3 as described previously (Wickersham et al., 2007; Osakada et al., 2011; Osakada and Callaway, 2013). SADΔG-GFP, SADΔG-mCherry, and SADΔG-BFP were recovered in B7GG cells with transfection with the corresponding genomic plasmid, pcDNA-SADB19N, pcDNA-SADB19P, pcDNA-SADB19L, and pcDNA-SADB19G. Viruses were amplified in B7GG cells in a humidified atmosphere of 3% CO_2_ and 97% air at 35°C and concentrated by two rounds of ultracentrifuge. The concentrated rabies viruses were titrated in HEK293t cells. The titers of the rabies viruses used in the present study were 5.0 × 10^8^ – 3.0 × 10^9^ infectious units/ml.

Adeno-associated viruses (AAV) were generated by the GT3 Core facility at the Salk Institute. AAV1-CMV-eGFP and AAV2-CMV-eGFP was generated at titers of 1.20 × 10^11^ – 5.13 × 10^12^ infectious units/ml. The viruses were stored at −80°C until use.

### Animals

This study was carried out in accordance with the accordance with the guidelines for care and use of animals by the Institutional Animal Care and Use Committee (IACUC) of the Salk Institute for Salk Institute for Biological Studies. The protocol was approved by the Institutional Animal Care and Use Committee (IACUC) of the Salk Institute for Biological Studies. Twenty mice (male and female) aged 3–4 months were used each having 1 to 2 injections (*N* = 23 injections total). The GCL was target with stereotaxic coordinates (Bregma +4.25 mm rostral/caudal, +1 mm medial/lateral, −0.6 mm to −1.5 mm dorsal/ventral) using a digital micromanipulator (Kopf, CA, USA). Stereotactic injections to the CA1 region were made relative to bregma (−3.25 mm rostral/caudal, + 3.25 mm medial/lateral, −4 mm dorsal-ventral). Borosilicate micropipettes were pulled on a Sutter P200 and 40–200 nl of virus were delivered to each bulb via pulsed injection from a picospritzer (Parker, OH, USA). Three different fluorescent reporters (GFP, MCherry, and BFP) in the G-deleted rabies virus were used for the experiments and all 3 labeled neurons extensively, including labeling of dendritic processes. For AAV viral vectors, either eGFP or dsRed were used as fluorescent reporters. Additionally, we used a Cholera-Toxin-β subunit tracer for some anterograde experiments.

### Histology

Three to 10 days after the viral or fluorescent reporter injection, animals were sacrificed and perfused with 4% paraformaldehyde (PFA) and brains were extracted and transferred to a solution of 4% PFA/30% sucrose (Padmanabhan et al., 2010). Coronal sections, 100 μm in thickness, were made of the mouse brain from the bulb to approximately Bregma −4.25 mm, allowing us to characterize inputs to the bulb. In 3 of the animals, damage while extracting the brain resulted in incomplete reconstructions of some regions such as hippocampus and occipital cortex. In 2 additional animals, tissue warped during perfusion, and though these were qualitatively to determine properties such as injection site size, number of labeled neurons etc., they were not used in calculating the relative distribution of feedback projecting cells from different brain regions.

### Imaging and Data Management

Images were acquired with an Olympus VS110 slide scanner (Tokyo, Japan). Complete scans of all coronal sections were done at 5 virtual *Z*-planes for each section. Each coronal section produced a stack of 5 ~30,000 × 50,000 pixel 16-bit images, which were then collapsed into a single maximum intensity projection. Each whole brain scan resulted in ~1 TB of data. For analysis and viewing, each individual section was sorted in a pyramid representation. For each representation

(1) G(x,y)= I(x,y)for level, l=0

(2) $$G_{l}\left(x,y\right)=\;\frac{1}{M^{*}N}\sum_{m=-2}^{M}\sum_{n=-2}^{N}G_{l-1}\left(x+m,\;y+n\right)$$

Such that G is the pyramid representation and I the original image where *M* = 2 and *N* = 2. Our resolution resulted in representations for l = 0–3.

### Image Dilation and Correlation

Individual coronal sections were aligned and alignment validated by correlation analysis (using custom functions in MATLAB (MathWorks, MA, USA). First, images were thresholded at different image places using previously described methods (Padmanabhan et al., 2010). This method simply finds an optimal threshold for binary images, or a series of binary image planes corresponding to individual objects of interest. To extent this to identify the boundaries of cells and tissues, we performed additional image processing. First, the Moore-Neighbor tracing algorithm was used to isolate tissue boundaries for each section as well as boundaries for individual neurons (Gonzalez et al., 2004). Boundaries where then convolved with a 2-dimensional Gaussian to minimize alignment sensitivity to pixel noise, and then a correlation coefficient between all pairs of sections was used to validate the alignment.

### Cell Finding

For each fluorescent image section, regions of interest were manually identified. Only label within these identified areas was used for analysis, thereby excluding background fluorescence. To identify neurons in an automated way, we defined an image X as a where x_n_ corresponds to the nth pixel:

(3) $$x=x_{1},x_{2},x_{3},\ldots x_{n}$$

We defined another vector y where y_n_ corresponding to the *n*th pixel:

(4) $$y=y_{1},y_{2},y_{3},\ldots y_{n}$$

such that

(5) $$y_{n}\{\begin{array}{c} =1\;if\;x_{n}>i\\ =0\;else\;x_{n}\;\leq i \end{array}$$

where *i* was a value selected manually for thresholding. Individual sections were thresholded using either a manually selected threshold value, or alternatively, using an optimal image thresholding method (Padmanabhan et al., 2010).

(6) $$\text{corr}\left(\text{x},\text{y}\left(\text{i}\right)\right)=\;\frac{\sum_{\text{j}=\text{i}}^{\text{m}}{\text{n}}_{\text{j}}*\left(\text{j}-\bar{\text{x}}\right)}{\sqrt{\left(\sum_{\text{j}}^{\text{m}}{\left(\text{j}-\bar{\text{x}}\right)}^{\text{2}}*{\text{n}}_{\text{j}}\right)*\left(\frac{\left({\text{N}}_{\text{m}}{\text{-N}}_{\text{i}}\right)*{\text{N}}_{\text{i}}}{{\text{N}}_{\text{m}}}\right)}}$$

where x is the original image and the thresholded image y at the threshold value (i-1) with j as the bit-depth of the image (for example, between 0 and 255 for an 8-bit image), n_j_ as the number of pixels with the value j and $\bar{\text{x}}$ as the mean pixel value of the image. In addition, N_m_ was the total number of pixels in the image and N_i_ was the number of pixels above the threshold value (i-1). We rewrote our correlation where x was the original image and y(i) was the image thresholded at a value of i. The optimal correlation:

(7) argmax t(m)=corr(x,y(m))

Thresholded images were then run through a series of image processing functions to identify retrogradely labeled neurons. To take thresholded images and segment individual neurons (which constitute multiple pixels), we first, performed a nearest-neighbor pixel grouping to assign contiguous pixels into neurons. Following this step, groups of pixels were sorted based on morphological properties, including the shape (how round to the neurons look, which was biased by the median filtering step) and the number of pixels within each group (which eliminated small pixels that corresponded to either processes or large groups of cells that are clumped together and cannot be easily segmented).

### Data Analysis

All analysis described in this manuscript, including tissue identification, was done with custom functions written in MATLAB. Unless otherwise noted, error bars are standard deviations.

## Results

**Figure 1:**
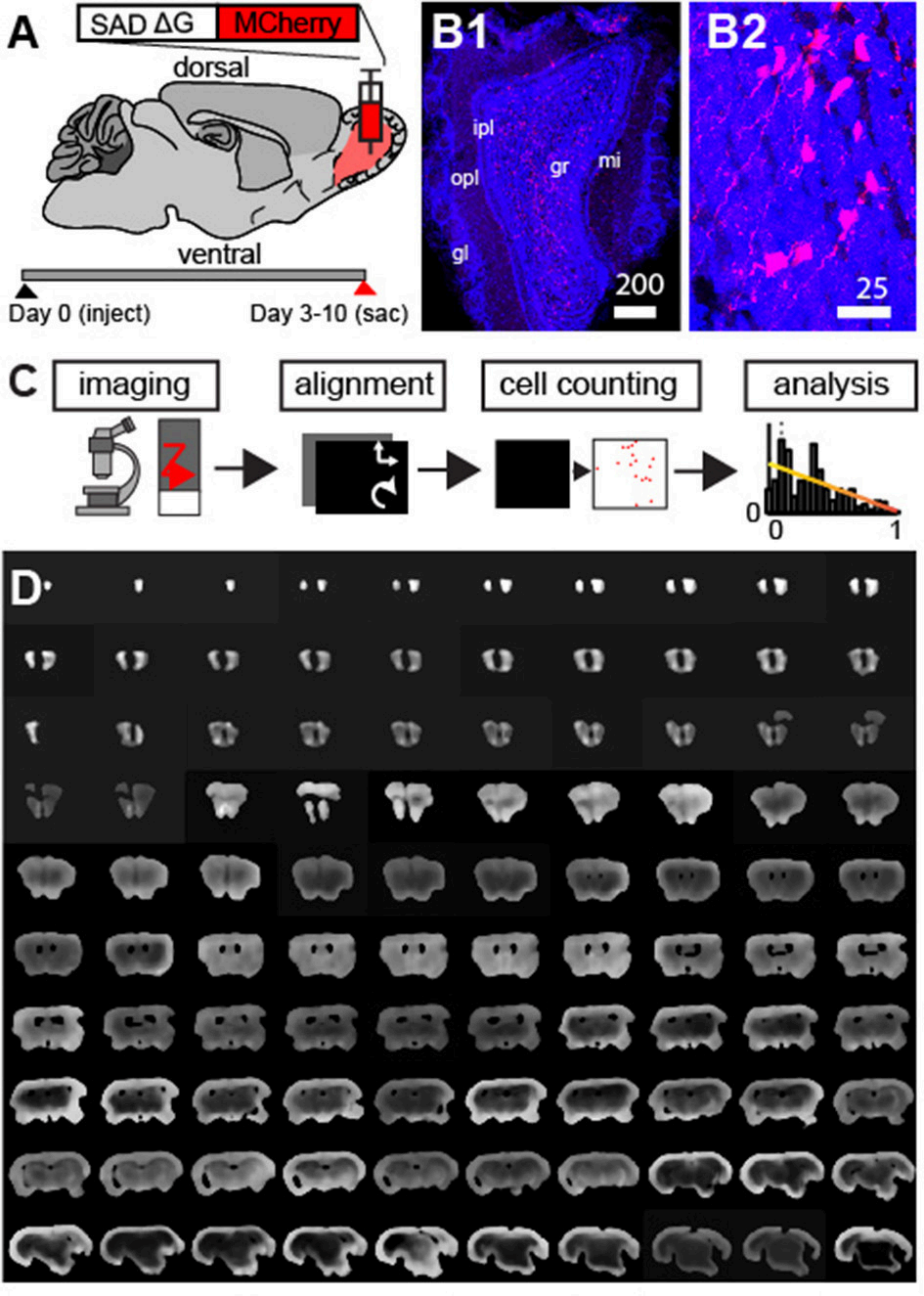
Modified g-deleted rabies tracing to identify feedback projection to the olfactory bulb. **(A)** Schematic outlining experimental design. **(B1)** Neurons labeled with mCherry rabies (red) and DAPI nuclear counter-stain (blue) at the injection site. Scale bar = 200 um. mi, mitral cell layer; opl, outer plexiform layer; ipl, inner plexiform layer; gl, glomerular layer; gcl, granule cell layer. **(B2)** Enlargement of neurons in **(B1)** shows clearly labeled neurons in the GCL. **(C)** Outline of approach involves automated fluorescence microscopy, coronal section alignment, automated cell counting, and subsequent analysis. **(D)** Example of coronal sections used for whole brain reconstructions.

A g-deleted rabies virus expressing a fluorescent reporter was injected into the granule cell layer (GCL) (Haberly and Price, 1978) of the MOB in adult mice (3–6 months of age, *N* = 20, Figure 1A). Retrogradely labeled cells infected with the virus did not express the rabies glycoprotein, thus fluorescence was confined only to neurons at the injection site (Figure 1B) and to those cells whose axonal buttons terminated at the site of the injection (Wickersham et al., 2007; Osakada and Callaway, 2013; Callaway and Luo, 2015). As feedback input to the glomerular layer (Petzold et al., 2009) and the mitral cell layer (Yan et al., 2008; Markopoulos et al., 2012) have been previously studied, we focused our analysis on the GCL. Following 3–10 days of viral infection (*N* = 4 at 3 days, *N* = 17 at 5–10 days), animals were sacrificed and coronal sections of the whole mouse brain were made. To characterize the spatial organization of centrifugal projections to the GCL, we established a whole-brain imaging and analysis method (Figure 1C). First, we collected data from ~160 to 180 100 μm sections constituting the mouse brain (Figure 1D, we did not section regions of the cerebellum and the most caudal regions of the brainstem).

**Figure 2:**
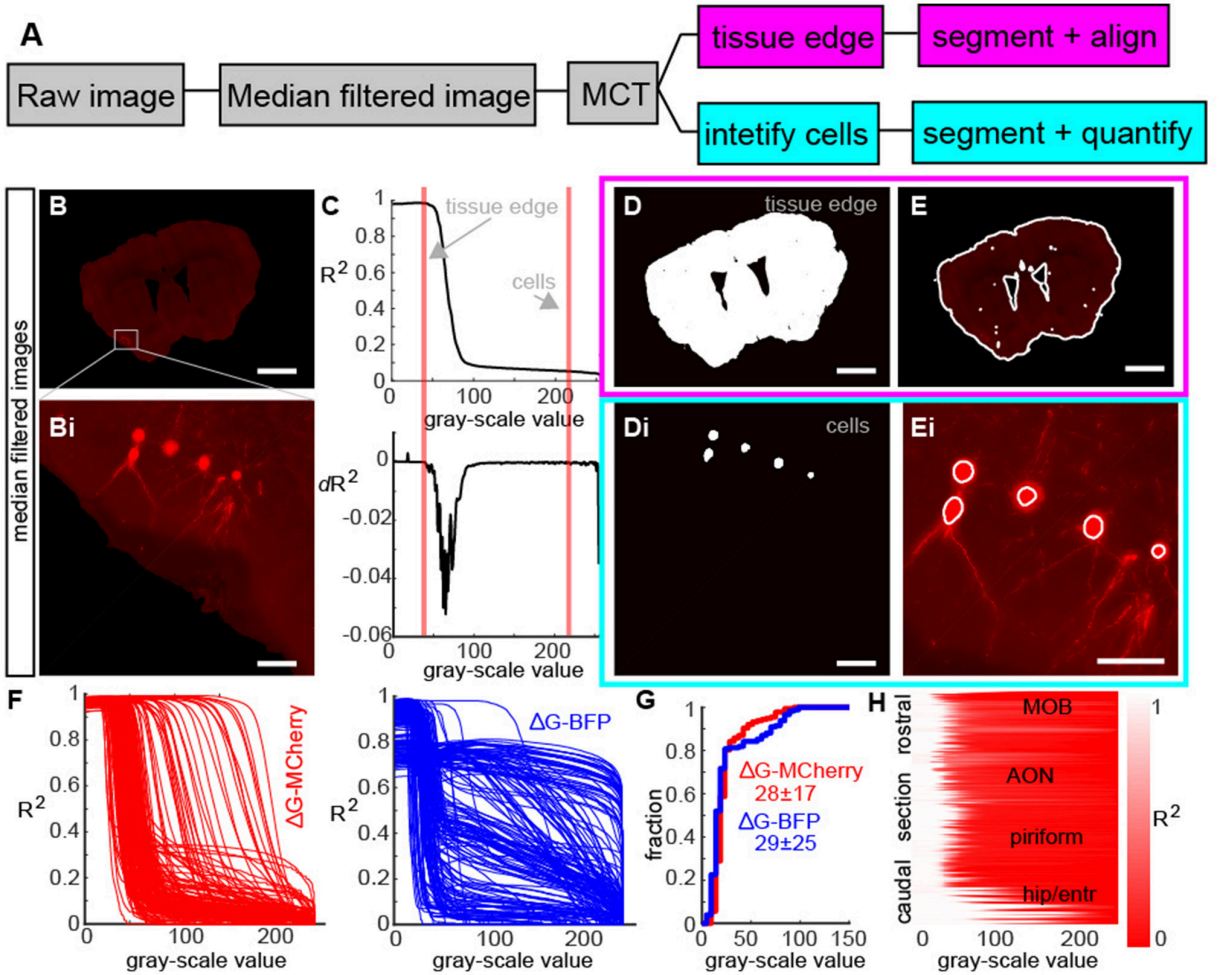
Image processing steps for whole brain reconstructions. **(A)** Flow diagram of image processing for identifying tissue structure and neurons. **(B)** Representative example of a median filtered fluorescence image section and **(Bi)** enlargement of retrogradely labeled neurons showing blurring of cell bodies due to filtering. (**C**, top) Correlation of a thresholded image to the median filtered image as a function of threshold value and (**C**, bottom) derivate of correlation curve. Red lines show transitions in the image statistics that correspond to the tissue edge which identifies the section boundaries and labeled cells. **(D)** Example of thresholded images from A at the values corresponding to the tissue edge and **(Di)** to retrogradely labeled neurons. **(E)** Outline of tissue edge and **(Ei)** individual cells shows that the algorithm identifies both the sections in the imaging and individual neurons. **(F)** Correlation curve (as described in **C**) for all sections in an experiment using G-MCherry (left) and G-BFP show the diversity of threshold functions across different sections and different viral constructs. **(G)** Cumulative histogram of threshold values selected for each section in **(F)** for G-MCherry and G-BFP highlight this diversity. **(H)** Correlation curves as a function of rostral-caudal axis shows a systematic variation from the olfactory bulb to the hippocampus and entorhinal cortex, reflecting the ability of the algorithm to segment sections and identify cells across the systematic differences in fluorescence associated with different sections.

In order to identify all retrogradely labeled neurons throughout the brain, we needed to perform two segmentation and identification tasks: isolate the tissue to delineate brain areas and identify the location of individual cell bodies (Figure 2A). To do this, we first median filtered the raw images (Figure 2B) to eliminate shot noise in our imaging, as well as to smoothen out the edges of the cell bodies (Figure 2Bi). Although this did reduce the ability to reconstruct individual dendrites (Figure 2Bi), it allowed us to rapidly segment individual neurons and the regions of the brain they inhabited. Next, we used the Maximum Correlation Thresholding (MCT) algorithm (Padmanabhan et al., 2010) which thresholds the image at each single gray-scale value and then calculates the correlation of the thresholded image to the original (Figure 2C, top). The curve represented the correlation of the thresholded image to the original image for each gray-scale value, and had distinct transitions points that were clear when we visualized the derivative of the correlation curve (Figure 2C, bottom). Changes in the correlation corresponding to the edge of the tissue (Figure 2C, red line) and the labeled cells (Figure 2C, purple line) were apparent in both the correlation curve and the derivative curve. While the MCT algorithm provides thresholded images corresponding to various objects (tissue edge, individual neurons, etc.), it does not identify which feature are important, nor does it segment these individual images into objects (such as the various boundaries of tissues, or neurons) that are defined by multiple contiguous pixels in an imaging plane. To address these issues, we first chose thresholding at these two different gray-scale values (Figure 2D), and using existing image segmentation algorithms (Padmanabhan et al., 2016) allowed us to define the tissue shape for each section (Figures 2D–E) as well as identify the individual cell bodies (Figures 2Di–Ei). As different traces have different levels of fluorescence corresponding to different objects (due to experimental variability like injection size, viral titer, cell type, and image parameters) we confirmed that the MCT algorithm produced different correlation curves (Figure 2F, left, red) reflecting differences in fluorescent pixel intensity across the various sections in a single mouse. The approach was robust to differences in pixel intensity across multiple fluorophores including GFP, RFP and, in this example Blue Fluorescent Protein (BFP, Figure 2F, right, blue curves). While the bit-depth for the maximum correlation was different across different experiments (Figure 2G), the shape of the correlation coefficient curve varied smoothly along the rostral caudal axis (Figure 2H), reflecting the overall decrease in mean fluorescence from the injection site in the bulb (where the density of labeled cells was high) to the caudal regions of the mouse brain.

**Figure 3:**
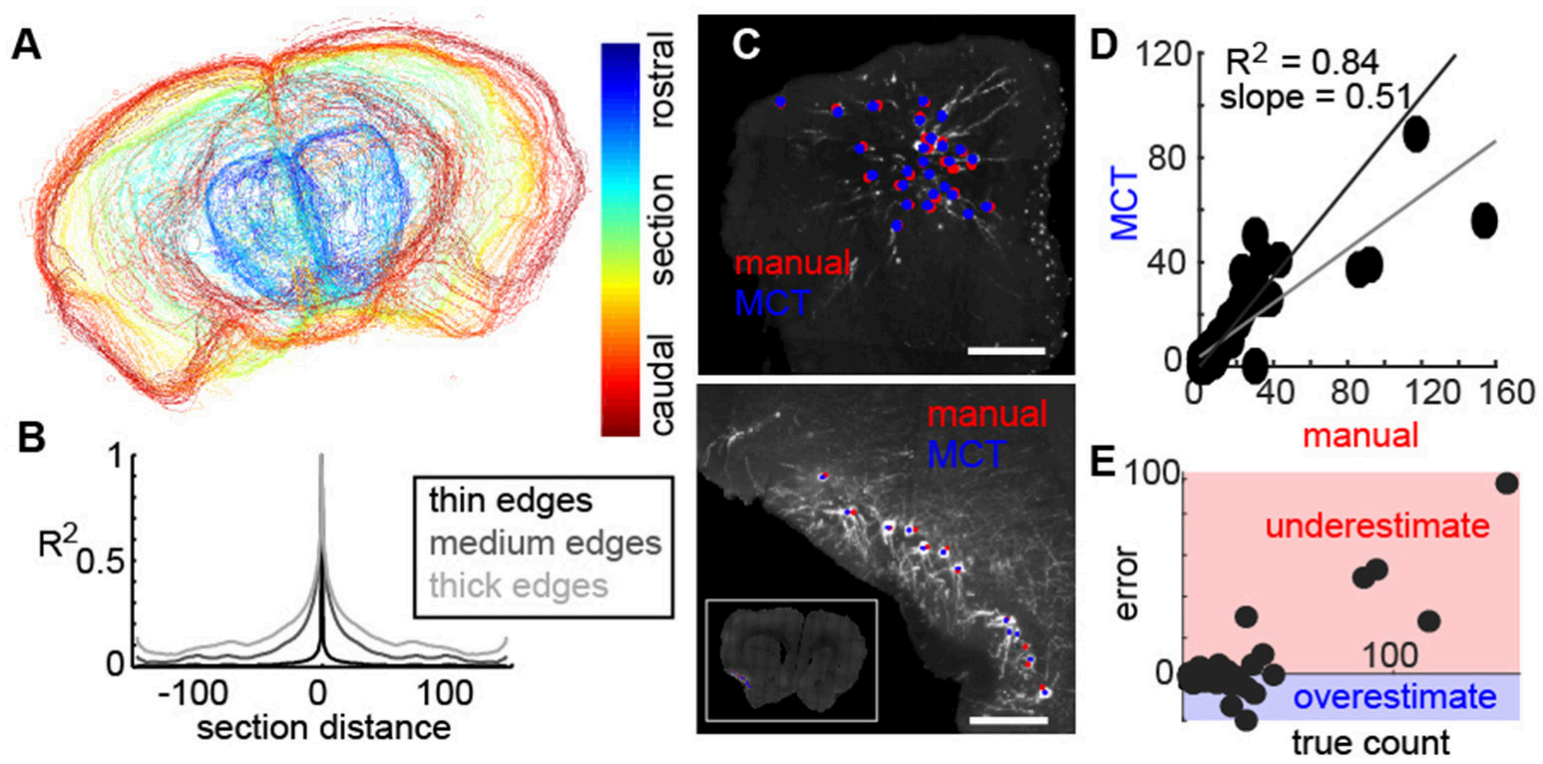
Fidelity of aligned tissue and identified neurons. **(A)** Representative example of sequential sections of the mouse brain identified using MCT. **(B)** Correlation of each section to the sections in front and behind with varying degrees of precision (as controlled by edge thickness) shows a tight alignment of individual coronal sections in the reconstruction. **(C)** Representative examples of neurons manually identified (red) and using the MCT algorithm (blue) near the injection site (top) and in a section with retrogradely labeled neurons. **(D)** Correlation of neurons identified by the MCT algorithm to the number of neurons identified by an expert human shows a high agreement (*R*^2^ = 0.84). **(E)** Error of MCT algorithm in identifying neurons as a function of neurons labeled in the section shows that the MCT. consistently underestimates the number of labeled cells when the label density is high, likely due to poor segmentation of neurons near the injection site and when cell clusters are densely packed.

Next, we wished to align the tissue and validate the image segmentation of cell bodies to assess the utility of the method. Using the thresholded images in 2D, we align section edges along the rostro-caudal axis (Figure 3A), which provided a 3D representation of the entire mouse brain. To determine the fidelity of this alignment, we calculated the correlation of each section to all adjacent sections (Figure 3B), with varying degrees of tolerance (as represented by the thickness of the edges of each section = gray lines). Correlation values were high in adjacent sections, but fell of dramatically, confirming that alignment of individual sections was tight (Figure 3B).

Following segmentation of the tissue, a segmentation was performed on the threshold images corresponding to labeled neurons which included shape analysis and cell size identification (See methods). The result of the algorithm appeared to be well-identified neurons. To determine the fidelity of the automated cell finder, in a subset of coronal sections from multiple animals, an expert used identified individual neurons manually and these were compared to the algorithm. Two representative examples of the olfactory bulb (Figure 3C, top) and the piriform cortex (Figure 3C, bottom) show neurons that were identified by the human user manually (red dots), and by the MCT algorithm (blue dots). In both examples, the automated cell finder identified the neurons that an expert human also identified in two distinct regions, with different fluorescent intensities, different neuronal morphologies, and different densities. When we quantified the accuracy of the MCT algorithm to the manual count, we found that across all comparisons, there was an *R*^2^ = 0.84, and this was significantly different from chance (Figure 3D, black line is the unit line, gray line is the best-fit for the data). When we examined the correlation between the manual expert and the MCT algorithm further, we found that underestimates occurred when the density of neurons being counted was high (Figure 3E, red area). These corresponded to regions at or near the injection site, where multiple neurons overlapped one-another and the algorithm had difficulty parsing them into individual cells. Despite this limitation, the 0.84 correlation of the algorithm to neurons identified by an expert human, allowed us to study the organization of feedback projecting cells to the bulb.

**Figure 4:**
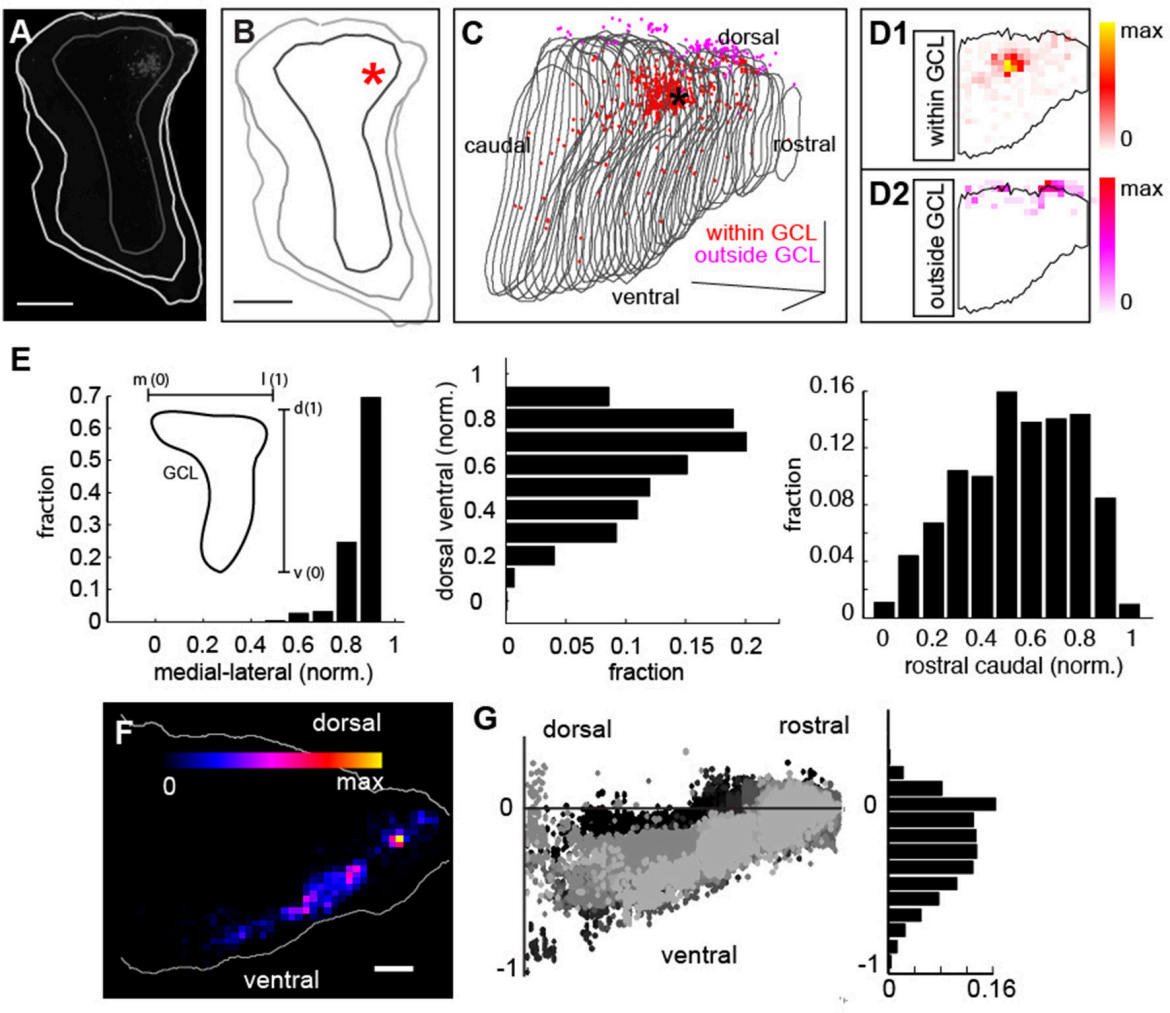
Distribution of injection sites and retrograde label throughout the mouse brain. **(A)** Coronal section of injection site with GCL border in dark gray and tissue border in light gray. Scale bar = 500 um. **(B)** Reconstruction of coronal section in **(B)** with injection site in asterisk. Scale bar = 500 um. **(C)** 3D reconstruction of GCL (gray lines correspond to each coronal section) viewed from a sagittal perspective within neurons at the injection site within the GCL in red and fluorescently labeled neurons outside the GCL in magenta. Scale bar = 500 um. **(D1)** Density of neurons at the injection site within the GCL (black outline) and **(D2)** density of neurons outside the injection site (black outline) but within the bulb. **(E)** Distribution of labeled neurons in the GCL for all experiments along the medial-lateral axis (left), and the dorsal-ventral axis (middle) and the rostral-caudal axis (right). The insert in **(E)** shows that the skew in the distribution is due to the skew in the morphology of the GCL. **(F)** Dorsal ventral density distribution of labeled neurons from 1 example experiment. **(G)** Dorsal-ventral scatter (normalized to injection site) for 10 experiments, (left) reveals that retrograde labeled cells are confined largely to the ventral regions of the mouse brain (right) corresponding to regions who trace their origins to archicortex.

We first analyzed the precision of our injections to the GCL of the bulb (Figures 4A,B). Boundaries were manually drawn in the tissue (light gray) between the inner plexiform layer (IPL) and the GCL (Figures 4A,B, dark gray) with the asterisk corresponding to the center of mass of the injection site (taken as the average of the Euclidian position of all the labeled neurons within the bulb). A 3D reconstruction of the bulb from this experiment revealed that although the injection site was confined to the GCL (Figure 4C, asterisk, red points correspond to labeled neurons within the GCL, gray lines correspond to the boundaries of the GCL for each coronal section), a small subset of neurons were also labeled outside the GCL (Figure 4C, magenta cells), likely due to label being taken up by neurites passing through the injection site and corresponding to mitral/tufted cells (Hovis et al., 2010; Padmanabhan and Urban, 2010). We assessed the ratio of labeled cells inside (Figure 4D, red cells) to cells outside the GCL but still within the bulb (Figure 4D, magenta cells) to determine the precision of our targeting (Figure 4D, black line in each corresponds to the GCL boundary). In this example, 84% of fluorescently labeled neurons were inside the GCL, similar to the percentage of neurons inside the GCL across all experiments, (80 ± 19%, *N* = 9).

When we examined the distribution of neurons within the injection site, we found that they were confined to the GCL (Figure 4E), with asymmetries in the density of label corresponding to the non-spherical shape of the GCL along the medial-lateral axis (Figure 4E, left). Additionally, our injections were slightly biased to the dorsal and caudal domains of the GCL as evidenced by the skewed distribution of the labeled cells along the dorsal-ventral axis (Figure 4E, middle) and the rostral-caudal axis (Figure 4E, right). While the representative example in Figure 4C shows a dorsal biased injection, additional injections labeled various regions of the GCL (Figure 4E). Although we observed no significant/systematic differences in the label density across position (Figure 4E), this does not rule out the possibility that such differences could exist. Importantly however, these distributions corresponded to the 3D volume of the GCL in our reconstructions (Figure 4C), further confirming the precision of our targeting. To determine the regions projecting to the GCL, we next examined the distribution of retrogradely labeled neurons throughout the mouse brain. A density map from the injection site in 4C (Figure 4F), and the distribution of label across 10 experiments for which we had reconstructions (Figure 4G, left), revealed that feedback-projecting neurons were almost entirely from ventral regions of the mouse brain (Figure 4G, right), predominantly archicortical in origin (piriform cortex, CA1, etc.) or neuromodulatory (HDB). As we saw label across these regions in an array of experiments (Figure 4G), feedback projections are likely to target both dorsal and ventral bulb.

**Figure 5:**
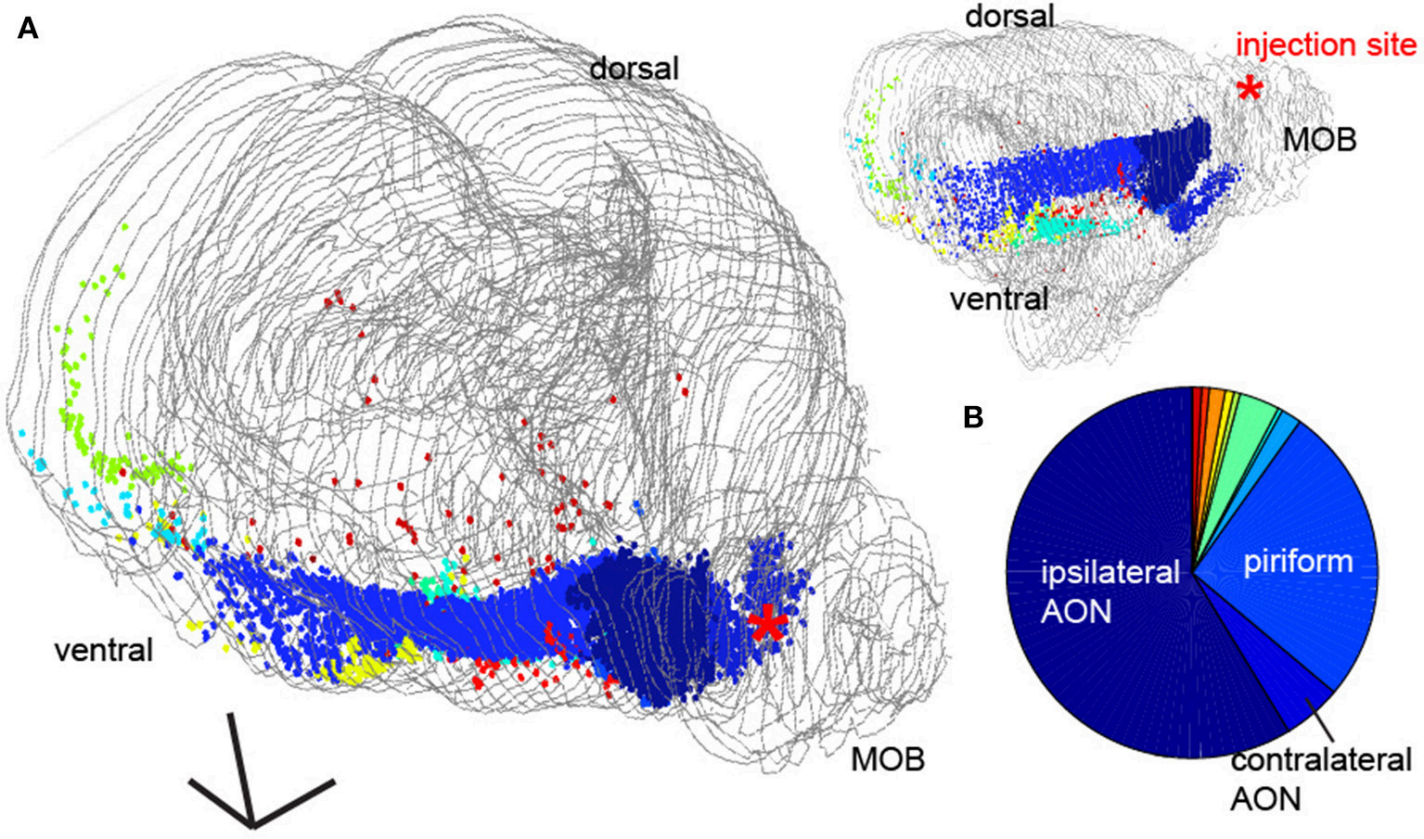
Area identity of feedback projections to the main olfactory bulb. **(A)** Whole brain 3D reconstruction (gray lines correspond to tissue edge for each coronal section) from 2 perspectives with fluorescently labeled neurons in different brain regions represented as points. Scale bar = 500 um. **(B)** Pie chart of the distribution of cells in different brain areas from the 3D reconstruction The three largest sources of feedback are highlighted.

To provide a more detailed description of the origins of the feedback projections, we aligned all of the brain sections to a mouse brain atlas (Paxinos and Franklin, 2004) and identified the areas corresponding to retrogradely labeled neurons (Figure 5A). We found labeled neurons in the both the ipsilateral (58%) and contralateral (5%) anterior olfactory nucleus (AON), the piriform cortex and the nucleus of the lateral olfactory tract (nLOT, 27.9%), the horizontal limb of the Diagonal Band (HDB, 3.5%), amygdala regions (1.4%), zona inserta (0.001%), the piriform-entorhinal cortex (0.001%) transition and in various nuclei of the hypothalamus (0.08%), consistent with previous reports (Figure 2F, Shipley and Adamek, 1984) as well as projections to the GCL directly from the entorhinal cortex (Figure 5A, light blue cells, 0.3%) and direct centrifugal input from the CA1 region of the hippocampus (Figure 5A, green, 0.75%) as had been hypothesized to exist previously in other species (de Olmos et al., 1978). We quantified the density of these projections from various regions (Figure 5B), and found that while the largest density of retrograde labeled cells came from major olfactory cortical areas (ipsilateral AON, contralateral AON = 63%), 10% of the retrogradely labeled neurons in the brain originated from areas that are not thought of as part of olfactory cortex.

Perhaps most unexpected were the retrogradely labeled neurons we found in the hippocampus and the entorhinal cortex [Figure 5A, light blue cells, and green cells (Leitner et al., 2016)]. Although some recent work has suggested that pyramidal cells in the CA1 region of hippocampus send axons directly to the bulb (Okuyama et al., 2016), it remains unclear if these are projections directly to the MOB or if these target the accessory olfactory bulb (AOB)(de Olmos et al., 1978; Shipley and Adamek, 1984).

**Figure 6:**
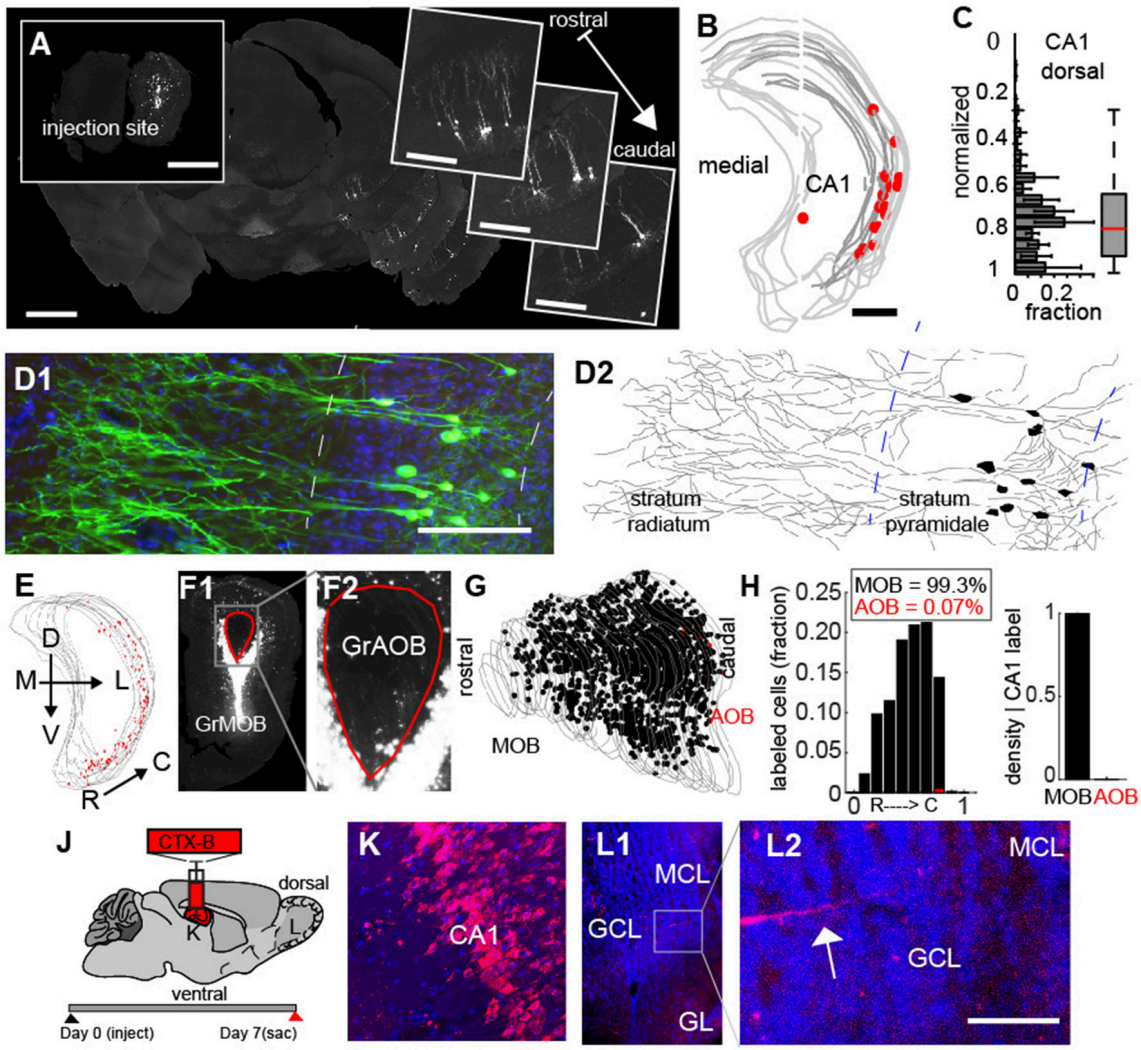
Monosynatic feedback projections from CA1 hippocampus to the main olfactory bulb. **(A)** Widefield fluorescent image of an example of feedback projecting neurons from CA 1 to granule cells in the olfactory bulb (insert). Scale bar = 500 um. **(B)** 3D-reconstruction of hippocampus/CA 1 boundaries with retrogradely labeled neurons (red). Scale bar = 500 um **(C)** Normalized dorsal-ventral distribution of retrogradely labeled neurons in CA 1. **(D1)** Confocal image of CA 1 neurons. **(D2)** Reconstruction of neuronal morphology from cells in **D1**. Scale bar = 200 μm. **(E)** Example of densly labeled neurons in vCA 1. Gray lines = CA 1 boundary. Red dots = retrograde labeled neurons. **(F1)** Injection site in the bulb corresponding to CA 1 reconstruction in **(E)**. GrMOB = Granule cells in main olfactory bulb (MOB). Red line corresponds to the accessory olfactory bulb (AOB) boundary. **(E2)** Enlargement of AOB from **E1**. GrAOB = Granule cells of AOB. **(G)** Reconstruction of all sections of the MOB injection from **(E1)**. Black dots = labeled neurons in MOB. Red dots = labeled neurons in AOB. Gray lines = granule cell layer boundary. **(H)** Distribution of labeled cells along the rostral-caudal axis (normalized) for MOB (black) and AOB (red) in left and ratio of MOB to AOB cells for densly labeled injection sites (right). CTX-B = cholera toxin B subunit. **(K,L)** Correspond to subsequent panels. **(J)** Schematic for anterograde label of axons projecting to the MOB from vCA 1. **(K)** CTX-B injection into CA 1. **(L1)** MOB with axonal arbors from CA 1 injection. **(L2)** Enlargement of G2 shows feedback processes from CA 1. Scale bar = 50 μm.

To address this, we determined the spatial location of the neurons projecting from CA1. First, we mapped the location of cells onto a 3D model of the CA region of hippocampus (Figures 6A,B) and found retrogradely labeled cells were confined largely to the ventral-lateral region (Figure 6C, *N* = 6), and could be identified as pyramidal based on their morphology (Figures 6D1, D2). As the rabies label filled dendritic processed, we were able to reconstruct multiple neurons, all of which had soma in the *stratum pyramidale*, apical dendrites projecting into the *stratum radiatum* and basal dendritic arbors in the *stratum oriens* (Figure 6D2). To the best of our knowledge, these are the only direct projections from the CA1 to a primary sensory area (Oh et al., 2014), and they are all the more interesting because they target neurons 2 synapses downstream from olfactory receptors in the sensory epithelium where odors are first detected. To ensure that these vCA1 pyramidal neurons were not retrogradely labeled because our injections accidently spilled over into the AOB, we examined in detail the injection site in the bulb for the experiments (Figure 6E, *N* = 6) where vCA1 cells were densely labeled. In these experiments, the MOB (Figure 6F1) was demarcated from the AOB (Figure 6F2). Neurons labeled in the injection site (Figure 6G, MOB cells in black, AOB cells in red) revealed that less that 1% of the fluorescent labeled neurons in the injection site (99.3% = MOB, 0.07% = AOB, Figure 6H) were in the granule layer of the AOB, further suggesting that projections from vCA1 did project directly to the GCL of the MOB. We further confirmed this direct projection by making injections of the anterograde tracer Cholera Toxin-β subunit conjugated to an Alexa 488/555 dye (Figure 6J) into the CA1 region (Figures 6J,K). Clear axonal projections in the GCL of the bulb (Figures 6L1, L2), providing complementary evidence of the link between CA1 and the olfactory bulb.

## Discussion

By combining a retrograde virus-labeling strategy with whole-brain 3D reconstruction methods, we describe the structure of centrifugal projections to the GCL of the bulb. First, from a methodological perspective, we provide an approach to segmentation of tissue and neurons that could be used more generally in a number of imaging applications. For instance, in light sheet microscopy application where voxel based approaches have been used (Ahrens et al., 2013), the strategy described here could afford additional insight into the dynamics of single neurons. Additionally, this method may be combined with brain clearing approaches (Liebmann et al., 2016) to reveal the spatial distributions of neurons within and across brain areas. Second, in addition to known projections from olfactory areas such as the AON and the piriform cortex and neuromodulatory areas such as the HDB, we identified direct CA1 pyramidal cell projections from the ventral/lateral region of the hippocampus and the entorhinal cortex directly to the bulb. Although these tracing methods cannot identify the cell types targeted by centrifugal projections to the GCL, which include granule cells, short axon cells, and the lateral dendrites of mitral/tufted (M/T) cells, they do suggest a diverse group of areas all send centrifugal projections to the bulb and could ultimately influence the neuronal activity of M/T cells in response to incoming stimuli. While this feedback and reciprocal connectivity has been identified previous in other species (Shipley and Adamek, 1984; Van Groen and Wyss, 1990) including the mouse (Mohedano-Moriano et al., 2012), and the findings of this study are in line with these previous descriptions, recent studies have overlooked these projections in detailing hippocampal connectivity in the mouse (Bienkowski et al., 2018). Additionally, our imaging method allowed us to identify the spatial pattern of cells across the brain (Figures 3–5) as well as provide a quantitative description of the relative density of cell projections from all of these feedback regions (Figure 5). Notably, we quantified that nearly 10% of projections come from regions other than olfactory cortex and piriform (Figure 5). Furthermore, that feedback from regions such as vCA1 and EC are pronounced across multiple experiments (Figures 5, 6) suggests that these circuits could play important roles in shaping odor representations. Although these regions constitute ~1% of the feedback projections we identified, they number of cells corresponds to the ratios observed in amygdala (1.3%) and ~1/3 of the cell density of feedback from HDB (3%). Other previous work has shown the role that cholinergic neurons from the basal forebrain play in shaping mitral cells responses (Rothermel et al., 2014), suggesting that even small number of connections, such as those from vCA1 and entorhinal cortex may have a functional role. Furthermore, as vCA1 also projects to piriform cortex, both monosynaptic and disynaptic feedback projections may pay direct and indirect roles across multiple timescales to influence the firing of mitral and tufted cells. Finally, while our method examines the feedback to the GCL, distinctions in between dorsal and ventral bulb (Kobayakawa et al., 2007) may emerge functionally based on asymmetries in the feedback. The functional role of these projections shall be an important focus of future experiments.

While M/T cells, the principal neurons of the bulb are responsive to odor stimulation, their activity can be modulated by other sources (Kay and Laurent, 1999), including centrifugal input, both from olfactory regions (Wesson et al., 2008; Boyd et al., 2012; Markopoulos et al., 2012) and from neuromodulatory centers (Rothermel et al., 2014). In addition to these known projections, we characterized additional feedback from the ventral region of CA1 hippocampus, whose spatial location may provide a hint of their functional role (Moser and Moser, 1998; van Strien et al., 2009; Fanselow and Dong, 2010). Coherence in the electrical activity of Local Field Potentials (LFPs) between the MOB and the hippocampus has been previously described (Martin et al., 2007), including oscillatory coupling in the beta (15–35 Hz) frequencies (Gourévitch et al., 2010), suggesting that these feedback circuits could play a role in LFP coherence between the two regions. Furthermore, ventral/lateral regions of CA1 are important for encoding stress responses (Henke, 1990) and, accordingly, feedback could carry this behaviorally relevant information to gate odor processing at the level of the bulb.

The limitations of adducing function from structure alone notwithstanding, our description of centrifugal projections suggests unique features of olfactory system. First, unlike other sensory systems, olfactory circuits are highly compact. They target the amygdala and cortex without a thalamic way point and receive direct feedback at the earliest processing stages from a variety of brain regions. Second, circuits tasked with encoding chemical sensation are tethered to evolutionarily old circuits such as those of the hippocampus. Finally, centrifugal feedback to the bulb is diverse.

Unlike other sensory modalities, like vision or audition, where stimulus features have clear equivalents in perceptual space, the link between chemical structure and odor perception remains an open question (Secundo et al., 2014). We propose that one reason for this difference is that the olfactory system has privileged access to the information about prior olfactory representations, the animal's behavioral state, and input from learning and memory systems at the level of the mitral and tufted cells in the bulb via direct centrifugal innervation (Restrepo et al., 2009; Gire et al., 2013). Consequently, coding at the bulb may be far less of a purely sensory operation and more about the interplay between chemical sensation and memory (Wilson and Stevenson, 2003).

## Author Contributions

KP conceived the project, designed, carried out the experiments and performed the analysis, and wrote the manuscript. FO provided reagents and assisted with experiments. AT and EK performed histology and imaging. FO, AT, and EK provided feedback on the manuscript. EC, FG, and TS provided assistance with project development and experiment design and contributed feedback to the manuscript writing and editing.

### Conflict of Interest Statement

The authors declare that the research was conducted in the absence of any commercial or financial relationships that could be construed as a potential conflict of interest.
